# *Mycobacterium malmoense* pulmonary infection in France: a case report

**DOI:** 10.1186/s13104-017-2753-z

**Published:** 2017-08-31

**Authors:** Simon Grandjean Lapierre, Mustapha Fellag, Célia Magan, Michel Drancourt

**Affiliations:** Aix-Marseille Université, URMITE, UMR CNRS 7278, IRD 198, INSERM 1095-IHU Méditerranée Infection, 19-21 Boulevard Jean Moulin, 13005 Marseille, France

**Keywords:** *Mycobacterium malmoense*, Non-tuberculous mycobacteria

## Abstract

**Background:**

*Mycobacterium malmoense* infections have frequently been reported in northern Europe since the late 1970s. Factors accounting for this geographically localized epidemiology remain poorly understood.

**Case presentation:**

We report the case of a 54-year old man concomitantly diagnosed with non-small cell lung carcinoma and *M. malmoense* pulmonary infection. We present detailed clinical, microbiological and radiological elements strongly arguing for *M. malmoense* true pathogenicity. Since *M. malmoense* infection has rarely been reported in France, we also provide elements of the epidemiological investigation and a literature review of potential acquisition and transmission pathways of *M. malmoense*. We detail therapeutic interventions and subsequent favorable evolution.

**Conclusion:**

*Mycobacterium malmoense* is a recognized respiratory pathogen for which routes of infection need to be better investigated.

## Background

Twenty years ago, the non-tuberculous mycobacterium *Mycobacterium malmoense* was described as a new species after being isolated from the respiratory secretions of four patients with pulmonary infections in the city of Malmö, Sweden [1]. Since then, very few cases were reported outside this endemic region. Cumulative reporting of *M. malmoense* infection cases allowed the recognition of two distinct associated clinical syndromes, namely pediatric isolated lymphadenitis and adult tuberculous-like pulmonary infection. Pulmonary infections most frequently occur among long-time smoking patients with chronic obstructive pulmonary disease but acute invasive or disseminated presentation may be seen in the context of significant immunosuppressive conditions such as hematologic malignancy and HIV [2, 3]. Atypical cases of septic arthritis, mycotic aneurysm and post-operative surgical site infection have also been reported [4–6]. As seen with other mycobacterial species, concomitant or subsequent *Aspergillus* spp. infection of pre-existent or mycobacterial disease induced pulmonary lesions may occur with *M. malmoense* [7].

Few cases of *M. malmoense* have been reported outside the endemic region and in particular, only eleven cases have been reported in patients living in France. We are reporting one more such case, questioning the sources of infection in this patient.

## Case presentation

In 2016, a 54-year-old man was referred to our institution with a newly established diagnosis of non-small cell lung carcinoma. Clinical evaluation revealed chronic cough, appetite loss and cachexia. Prior to initial pulmonary bronchoscopy investigation, two consecutive tomodensitometry imaging studies had shown left hilar consolidation and right upper lobe cavitary lesions to be stable in time over a 1-year period. His past medical history included chronic obstructive pulmonary disease, chronic hepatitis C, associated cryoglobulinemia and lymphoma for which he had received chemotherapy and cervical radiation therapy in 2003. He was a chronic alcohol, tobacco and cannabis drug user and took no regular medication.

Prior to chemotherapy initiation, mycobacterial culture performed on the initial diagnostic bronchoalveolar lavage revealed the presence of *M. malmoense*. During the following 3-month period, *M. malmoense* was repeatedly isolated from six sputum and bronchial aspiration control specimens. According to local procedures, isolates were recovered from BACTEC™ MGIT™ (Becton–Dickinson, New Jersey, United States) radiometric mycobacterial broth culture system with incubation at 37 °C for an average of 25 days. Identification to the species level was confirmed by mass spectrometry and RNA polymerase ß-subunit coding *rpoB* gene sequence analysis per local procedures [8, 9]. The *rpoB* sequence was 612-bp long and shared 99.8% similarity with *M. malmoense* ATCC 29671 strain (GenBank Accession No. GQ153314). The second closest mycobacterium species was *Mycobacterium palustre* sharing only 571/612-bp 93.3% (GenBank Accession No. HM022210). Broth dilution antimicrobial susceptibility testing was performed per published Clinical Laboratory Standard Institute guidelines [10] and showed the isolate to be in vitro susceptible to rifampicin, clarithromycin, streptomycin and amikacin; and in vitro resistant to isoniazid, ethambutol, ofloxacin, doxycycline, tigecycline and linezolid.

Detailed epidemiologic and potential environmental exposure history revealed the patient had been incarcerated in France in 2016 and regularly ate both fox and squirrel bush meat. He reported having exceptionally eaten MukTuk, a traditional Inuit and Chukchi meal of frozen whale skin and sub-cutaneous adipose tissue and having been in close contacts with a friend and his dog upon their return from Scotland [11].

A treatment of isoniazid 5 mg/kg/day, rifampin 10 mg/kg/day, ethambutol 15 g/kg/day and clarithromycin 500 mg bid was initiated to cover both tuberculosis and *M. malmoense* infection. On control imaging following both antimycobacterial treatment and chemotherapy initiation, superior lobes consolidated and cavitary lesions had regressed. Clinical evolution was also favorable. On the other hand, nodular and reticulonodular infiltrates in lower lobes were progressing. These discordant radiologic findings were interpreted as ongoing infection. Extended microbiologic workup was repeated but unrevealing except for an additional *M. malmoense* positive culture.

Upon submission of this paper, the patient was still alive, undergoing his chemotherapy treatments and receiving full antimycobacterial therapy without side effects.

## Discussion

*Mycobacterium malmoense* infection has rarely been reported in France as only eleven such cases have been retrieved in our literature review [12–22]. We report on the twelfth case, firmly documented by the isolation of six different isolates from distinct and consecutive clinical samples collected from the patient here described. All the isolates have been identified with certainty using mass spectrometry and DNA sequencing in the presence of appropriate controls [8, 9]. Moreover, *M. malmoense* had not previously been isolated in our laboratory and was not handled in our laboratory at the time specimens were obtained from the patient, rendering in-laboratory contamination not possible.

*Mycobacterium malmoense* was interpreted as being responsible for initial clinical and persistent radiological features in this patient and he therefore received appropriate antibiotic treatment [23]. In the context of cachexia, poor residual pulmonary function and imminent chemotherapy initiation, our patient was empirically treated with a combination of four effective drugs. *M. malmoense* in vitro antimycobacterial susceptibility testing results and in vivo treatment outcomes were frequently shown to have poor correlation [24]. In 1999, based on in vitro synergistic activity and prior case series clinical data, the British Thoracic Society recommended a 2-year rifampin and ethambutol therapy for *M. malmoense* pulmonary diseases and proposed the use of surgical resection as an adjunctive approach for unilateral disease in patients with poor tolerance or response to medical treatment [25]. In 2000, a randomized controlled trial was performed on 223 patients with non-tuberculous mycobacteria (NTM) pulmonary disease including 106 *M. malmoense* infections [26]. Patients were randomized as receiving a two-year course of rifampin and ethambutol alone or rifampin, ethambutol and isoniazid combination therapy. After up to a 5-year follow-up period, no difference was observed between groups on deaths and microbiological failures outcomes. Besides recommended first line antimycobacterial agents, macrolides and fluoroquinolone have occasionally been used in combination therapy with variable outcomes [27]. The most recent published guidelines on the treatment of non-tuberculous mycobacteria from the American Thoracic Society and the Infectious Disease Society of America reinstated these same recommendations [23].

**Fig. 1:**
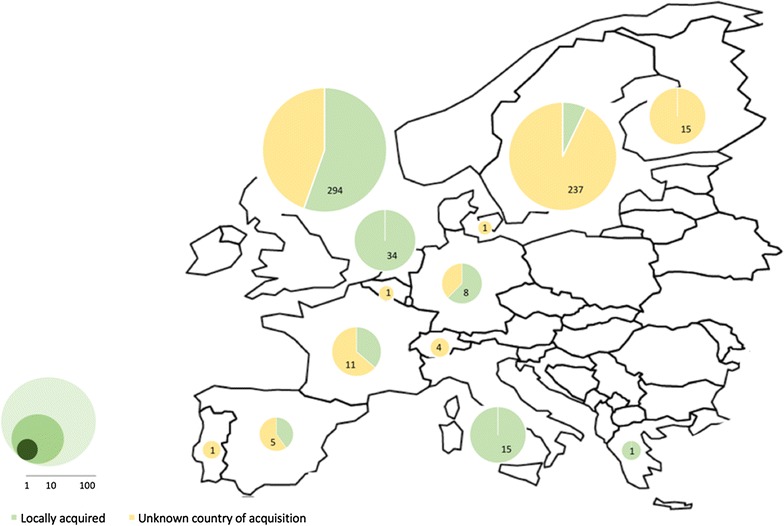
Reported human cases of *M. malmoense* infection in Europe. Literature review including case reports, case series and diagnostic laboratory positive culture series with associated clinical data. *Green* locally acquired infections, *yellow* unknown country of acquisition, total number of cases

A disproportionately high incidence of *M. malmoense* lymphadenitis and pulmonary infections in United Kingdom and Northern Europe was first reported in two case series of the 1980s [28, 29]. This epidemiologic trend was then confirmed by a large-scale 3-year retrospective study from 1990 to 1993 [30]. More than 25 years later, despite a wide distribution of accurate diagnostic tools and recognition of the species as a human pathogen, this geographic predominance persists (Fig. 1) [31]. In 2001, an epidemiologic investigation using restriction fragment length polymorphism molecular typing on 79 *M. malmoense* clinical isolates along with detailed patients’ demographic data failed to demonstrate patient to patient transmission [32]. Another large-scale epidemiologic study reviewing every diagnosed case in northern United Kingdom between 2000 and 2005 was also unable to identify space–time clusters of cases [33]. Even in the context of documented strong epidemiologic link between two cases with smear-positive sputum, molecular epidemiology infirmed human to human transmission [34]. This geographic distribution of disease is still poorly understood but available data do not support human to human transmission or common environmental infection source.

Reported animal infection cases include disseminated and skin infections in cats in Finland and United Kingdom [35, 36] and lymph node infections of cattle Northern Ireland and Hungary [37, 38] and pork in Netherlands [39]. These data and those from animal model experimental studies reveal that multiple mammal species are either vulnerable hosts or healthy carriers and therefore potential vectors of *M. malmoense*. Interestingly, animal and human reported cases have the same geographic distribution which could indicate a common environmental source or animal to human transmission. However, to our knowledge, human acquisition of *M. malmoense* from an animal or environmental source has never been reported either.

Mycobacterial host-adaptation has been correlated with potential ecological niches determinants such as immediate environment salt concentrations for other NTMs [40]. Environmental studies performed in Finland demonstrated that peatland soils and surface waters such as those from Scotland and the United Kingdom present optimal physico-chemical proprieties for the growth of *M. malmoense* among other NTMs [41, 42]. Our patient did not report being in contact with such environments on an exceptional or regular basis but clearly reported having been in contacts with a living animal and eating animal product from these geographic regions.

## Conclusion

This case is highly interesting since it documents *M. malmoense* infection in a patient living in a low-incidence country where no environmental or animal isolates have been described. Whether infection was acquired through respiratory or digestive route and whether human to human or human to animal transmission occurred could not be established. Other case reports such as the one of a 53-year old Canadian woman with previously normal lung function but chronic intestinal inflammation due to Crohn’s disease support the hypothesis of a digestive route of infection [43]. Moreover, *M. malmoense* has previously been isolated in human stools and this infection pathway is well documented for other mycobacterial species [43–45].

Epidemiology studies linking genetically identical human and environmental isolates in space and time are still needed to better understand *M. malmoense* routes of infection.

## Abbreviation

NTM: non-tuberculous mycobacteria
